# Unconventional Transcriptional Response to Environmental Enrichment in a Mouse Model of Rett Syndrome

**DOI:** 10.1371/journal.pone.0011534

**Published:** 2010-07-12

**Authors:** Bredford Kerr, Pamela A. Silva, Katherina Walz, Juan I. Young

**Affiliations:** 1 Department of Biology, Centro de Estudios Científicos, Valdivia, Chile; 2 Department of Biochemistry, Universidad Austral de Chile, Valdivia, Chile; 3 CIN (Centro de Ingeniería de la Innovación del CECS), Valdivia, Chile; 4 Department of Human Genetics, John P. Hussman Institute for Human Genomics, Miller School of Medicine, University of Miami, Miami, Florida, United States of America; National Institutes of Health, United States of America

## Abstract

**Background:**

Rett syndrome (RTT) is an X-linked postnatal neurodevelopmental disorder caused by mutations in the gene encoding methyl-CpG binding protein 2 (MeCP2) and one of the leading causes of mental retardation in females. RTT is characterized by psychomotor retardation, purposeless hand movements, autistic-like behavior and abnormal gait. We studied the effects of environmental enrichment (EE) on the phenotypic manifestations of a RTT mouse model that lacks MeCP2 (*Mecp2* ^−/y^).

**Principal Findings:**

We found that EE delayed and attenuated some neurological alterations presented by *Mecp2* ^−/y^ mice and prevented the development of motor discoordination and anxiety-related abnormalities. To define the molecular correlate of this beneficial effect of EE, we analyzed the expression of several synaptic marker genes whose expression is increased by EE in several mouse models.

**Conclusions/Significance:**

We found that EE induced downregulation of several synaptic markers, suggesting that the partial prevention of RTT-associated phenotypes is achieved through a non-conventional transcriptional program.

## Introduction

Rett Syndrome (RTT, OMIM # 312750) is a severe neurodevelopmental disorder constituting one of the leading causes of mental retardation and autistic features in females [1]–[3]. It affects approximately 1 in every 10,000 to 15,000 live female births [4]. Classic RTT patients are characterized by apparent normal development for the first months followed by developmental stagnation and regression. In the regressive phase, purposeful hand movements and any acquired speech are lost, brain growth decelerates, and ataxia, seizures, autonomic dysfunction and autistic features develop.

Mice null for *Mecp2* (*Mecp2* ^−/y^) exhibit neurological dysfunction that ends in premature death at 6–18 weeks of age, depending on genetic background [5]–[7]. *Mecp2* ^−/y^ mice have abnormal gait and movements, hypoactivity, hypotonicity, tremor, hindlimb clasping, and irregular breathing. This phenotype manifests at 4–6 weeks and leads to weight loss followed by death. Neurons of *Mecp2* null mice are smaller, more compactly arranged than wild type neurons [5] and present functional impairments. *Mecp2* ^+/−^ females develop a similar phenotype but with later onset.

Conditional deletion of *Mecp2* in either embryonic or postmitotic neurons results in a phenotype similar to complete knockout [5], [6], suggesting that RTT is caused by dysfunction of brain neurons. Additional support for this idea came from experiments in which the phenotypes of the *Mecp2* null mice were rescued by neuronal expression of transgenic *Mecp2* [8].

Evaluation of the features of RTT suggests that the basis of this disease could be an alteration of neuronal plasticity and studies of RTT mouse models support this suggestion. Long-term potentiation is reduced in cortical slices of mice mutant for *Mecp2* [9], [10] and loss of MeCP2 in mice results in a decrease in the total excitatory synaptic drive, an increase in the total inhibitory drive and an altered number of glutamatergic synapses [11]–[13]. Expression of BDNF, DLX5 and FXYD1, proteins that mediate neural plasticity have been shown to be altered in brains of RTT patients and MeCP2 mutant mice [14]–[19].

It has been therefore hypothesized that the RTT-like phenotype exhibited by *Mecp2* null mice is amenable of modulation by utilizing stimuli capable of eliciting neural plasticity. The paradigm most widely used to assess environmental-induced plasticity is called environmental enrichment (EE), and consist of a combined social and inanimate stimulation. EE attenuates neurological deficits associated with experimental brain injury [20] and delays disease onset and slows progression in mouse models of Huntington [21], [22] and Alzheimer's disease [23].

The effects of EE on mouse models of RTT have been analyzed with variable findings depending on the mice and enrichment protocol used. Kondo et al. [24] found EE-induced motor improvement in *Mecp2* ^+/−^ female mice but not in *Mecp2* ^−/y^ males, whereas Nag et al. [25] did find motor performance amelioration in *Mecp2* null male mice. In any case, the mechanisms underlying the beneficial effects of EE on RTT-like phenotypes have not been completely dilucidated.

Here we report that EE induces phenotypic amelioration in a RTT mouse model trough a “non canonical” transcriptional response.

## Methods

### Animals

In this study we used the Rett Syndrome mouse model generated by the laboratory of Adrian Bird [(strain *Mecp2^tm1.1Bird^*), Wellcome Trust Centre for Cell Biology, Institute of Cell and Molecular Biology, University of Edinburgh, Edinburgh, UK] [5], [6]. The mice we used were male mice (dubbed *Mecp2* ^−/y^) on a 129S1/SvImJ (N6) or F1s of a cross between 129/SvJ *Mecp2^−/^* ^+^ female and C57BL/6J males. Mice of the 129S1/SvImJ background were used for all determinations with the exception of the repeated anxiety test in which mice on the F1 background were used. Animals were kept in an animal room under SPF conditions at a room temperature of 20±2°C, in a 12/12 h light/dark cycle with free access to food and water. All the experiments were approved by the Centro de Estudios Científicos Care and Use Committee.

### Housing conditions

Three-week-old *Mecp2^+/y^* and *Mecp2^−/y^* mice were randomly allocated to either standard condition (SC) or environmental enrichment (EE) cages for 14 days. After this period of EE, mice were placed back into normal cages. SC include small group housing (4 mice per cage) in standard cages (30×15 cm) and provision of normal feed, water and bedding, whereas the cages of the EE groups (8 mice per two-connected 30×30 cage) contained a variety of cardboard, paper and plastic objects of a diversity of colors, that were changed daily. Food pellets were buried in the bedding in varied places. Additionally, mice had access to a free-running wheel to stimulate physical activity. Both types of cages were located in the same room, in ventilated racks.

### Phenotypic Testing and Behavioral Overview

All behavioral testing was done on age-matched male mice. Routine observation for RTT-like symptoms such as clasping and stereotypic hand movement, body weight determination and survival were performed at weekly intervals starting from 3 weeks of age. The level of severity of clasping was determined according to an arbitrary scaling of clasping (from 0 to 3) based on how fast mice clasp their feet together when picked by the tail and also on their capability of releasing the posture. 0 (no clasping), 1 (reversible clasping), 2 (delayed but irreversible) and 3 (immediate and irreversible). At 7 weeks of age, each mouse was subjected to a battery of behavioral tests performed always in the same order: elevated plus maze, open field and elevated beam test. For all experiments the data were presented as mean ± SEM. Statistical significance was set at a minimum of p<0.05. Survival analysis was conducted by means of a Kaplan–Meier survival analysis.

### Footprint Ink Test

To obtain footprint tracks, the soles of the four paws were dipped in ink and the mice were allowed to walk down an enclosed runway lined with white paper. The resultant ink footprints were analyzed by measuring the length of three strides for each hind leg and the hind-base width (the distance between the right and left hind-limb strides) from the middle portion of each run. Mean values were used for statistical analysis.

### Elevated Plus Maze

Mice were placed in the center of a cross-shaped maze elevated 45 cm from the floor with two open and two closed arms. The behavior of the mice was observed and the time spent in either the closed or open arm or in the center of the maze was recorded.

### Motor Abilities

Motor abilities were tested by the elevated beam test, which consists of two elevated platforms that are enclosed by walls in every side except in the side that connects them through a 70 cm long dowel of 0.7 cm radius. Before testing, mice were placed on one of the platforms and allowed to habituate for 1 min, then moved to the opposite platform for another min. Thereafter, mice received a short training, placing them on the dowel 10 cm away from one of the platforms. Only those mice that reached the platform in the first 60 sec were further assessed. Next, mice were placed in the middle of the dowel and the time of first arrival, the total number of arrivals and the number of falls were recorded.

### Real-time RT-PCR

Total RNA from hypothalamus and cortex were extracted with Trizol (Invitrogen, Calif.) according to the method provided by the manufacturer. RNA was treated with DNase I (Epicenter, WI, USA) for 30 min at 37°C, quantitated by measuring absorbance at 260 nm and stored at –80°C until used. RNA (2.5 µg) was reverse transcribed at 42°C for 60 min by using oligo dT primers and Tetra cDNA synthesis kit (Bioline Inc, MA, USA) to synthesize single stranded cDNA. PCR mixtures were prepared with Quantimix Easy Syg Kit for Real Time DNA amplification and quantification (Biotools, Spain). Quantitative Real-time RT-PCR amplifications were performed in triplicate from 25 ng of cDNA using the Rotor-Gene 6000 (Corbett, Australia) in a total volume of 10 µl, each reaction containing 1 µl of diluted cDNA. The results were analyzed with the Rotor-Gene 6000 Series Software 1.7 (Corbett) and all values were normalized to the levels of the GAPDH mRNA. Primers used were the following: for Syp (XM_125375) forward 5-GGAGTTTGAGCAGTGGGTGT-3 and reverse 5-GTGGGGTGGAATCAGGAGTA-3, Stx1 (NM_016801) forward 5-ACAAAGGAGGAACTGGAGGA-3 and reverse 5-GATGCTCTGCTCAATGCTCT-3, Syt9 (AB026802) forward 5-CATCGACCAGATCCACTTGT-3 and reverse 5-TCGTTTCCTACTTGGCACAC-3, Syn1 (NM_013680) forward 5-TTTTGTGGTCTGCATCTTCC-3 and reverse 5-ACTCTTCTCTTGGGGGTTCA-3, PSD95 (NM_007864) forward 5-GACGCCAGCGACGAAGAG-3 and reverse 5-CTCGACCCGCCGTTTG-3, Sgk-1 (NM_011361) forward 5-GATGGGCCTGAACGATTTTA-3 and reverse 5-TGCCCTTTCCGATCACTTTC-3, Igf1 (NM_184052) forward 5-TTGCGGGGCTGAGCTGGTGGAT-3 and reverse 5′-GCGGGCTGCTTTTGTAGGCTTCA-3, Igf-bp (NM_010065) forward 5-TGCCGCAGAGAAATGGAGGAC-3 and reverse 5-AGGGCGGCACTGCTTCTTCT-3, Bdnf (NM_007540) forward 5-CAGTGGCTGGCTCTCTTACC-3 and reverse 5- TGCTGCCATGCATAAAACAT-3, Grin2b (NM_008171) forward 5-ACGGCAGCAAATCCTACTTCT-3 and reverse 5-ACCACTGGCTTATTGGTGACA-3, Gria (NM_008165) forward 5-AAGAACGGTCAGAGGTGGTT-3 and reverse 5-AGAAGGCAGAAGGTGACACA-3, Gap43 (NM_008083) forward 5-GAGGAGAAGAAGGGTGAAGG-3 and reverse 5-GACGGGGAGTTATCAGTGGT-3, Nefm (NM_008691) forward 5-AGCTGGGTGATGCTTACGAC-3 and reverse 5- AGCTGCACTTGAGCCTTCTC-3, Dyn1 (NM_010065) forward 5-TGGTGCATTGCCTGGGTGGTGTG-3 and reverse 5-CCGATCGGCTGGCAGTTGGACAG-3, N-Cadh (NM_007664) forward 5-CCAGCAGATTTCAAGGTGGAC-3 and reverse 5-TTACAGCTACCTGCCACTTTTC-3, Arc (NM_018790) forward 5-AGGAGTCAGTTGAGGCTCAGCAAT-3 and reverse 5-TCATGTGGTTCTGGATCTGGGACA-3, Crh (NM_205769) forward 5-CAGAGCAGTTAGCTCAGCAAGCT-3 and reverse 5- GGCCAAGCGCAACATTTC-3 and GAPDH (NM_008084) forward 5-ACCCAGAAGACTGTGGATGG-3 and reverse 5-CACATTGGGGGTAGGAACAC-3.

## Results

### Effects of EE on the phenotype of a RTT mouse model

With the objective of studying the molecular correlate of the environmental enrichment–induced amelioration of symptoms exhibited by RTT mouse models, we subjected mice lacking exon 3 and the coding region of exon 4 of *Mecp2* (*Mecp2* ^−/y^ [5], [6]) to stimulating environmental conditions. We continually exposed 3 week-old *Mecp2* ^−/y^ and *Mecp2* ^+/y^ mice to an enriched environment for 14 days. We chose to initiate EE at this age since it has been reported that starting EE at weaning ameliorates some RTT features exhibited by different RTT mouse models [24], [25] and also because this enrichment protocol elicited a significant enhancement in cognitive abilities in wild type mice in our hands (Morris water maze, data not shown). The enriched environment consisted of two cages interconnected by a dark tunnel. Inside the cages we placed a variety of objects that were changed daily and a running wheel. The food was placed in different locations inside the cage and sometimes was buried in the bedding material. Social stimulation was also provided by the cohabitation of 8 mice instead of the standard 4 mice per cage.

At 3 weeks of age *Mecp2^−/y^* mice did not show any overt phenotype, but a week later they could be easily recognized by their altered gait, reduced body weight, tremors and hindpaw clasping, precluding us from being completely blind to the genotypes when performing the experiments. Because of this, we were able to notice that *Mecp2^−/y^* mice housed in enrichment cages (*Mecp2^−/y^*-EE) displayed an attenuated phenotype. It was visually evident that the movements of *Mecp2^−/y^* mice living in EE cages were more rapid and coordinated than their mutant littermates housed in control cages. We also observed that the development of paw clasping, a characteristic feature of *Mecp2^−/y^* mice commonly presented by mice with neurological problems, is delayed in approximately one week in the *Mecp2^−/y^*-EE in comparison to *Mecp2^−/y^*-SC mice. Home cage observations indicate that *Mecp2^−/y^*-SC mice displayed hindpaw clasping of a severity level arbitrarily scored as 1–2 at 4 weeks of age, and a week later it progresses to 2–3. Instead, the hind paw clasping developed by *Mecp2^−/y^*-EE mice was not detectable at 4 weeks of age (levels of severity 0) but started at 5 weeks following the same course of the *Mecp2^−/y^*-SC (level 1–2 on week 5 progressing to level 2–3 at week 6 of age).

At the end of the enrichment period, we evaluated more systematically its effect by a phenotyping screen that included determination of life span, physical and behavioral characterization.

**Figure 1:**
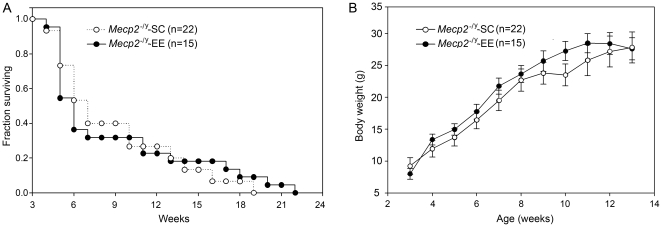
Environmental Enrichment does not modify life span or body weight of *Mecp2^−/y^* mice. A, Kaplan-Meier plots showing the survival of *Mecp2^−/y^*-SC (open circles) and *Mecp2^−/y^*-EE (closed circles) since the initiation of the enrichment period. B, Growth curves of mice showed in A. The body weights of *Mecp2^−/y^* mice, irrespective of housing conditions, dropped abruptly after 14 weeks and were therefore not included in the graph. Data represent mean ± SEM. The total number of animals in each group is shown (*n*). No significant differences were found between *Mecp2^−/y^*-SC and *Mecp2^−/y^*-EE according to ANOVA tukey test.

Comparison of life span of *Mecp2^−/y^* mice subjected to environmental enrichment (*Mecp2^−/y^*-EE) or to standard conditions (*Mecp2^−/y^*-SC) showed that life expectancy (similar 0.5 probability survival rate, approximately 6 weeks) was not modified by EE (figure 1A).

The enriched environment includes free access to a running wheel and we observed that *Mecp2^−/y^* mice spent a considerable amount of time exercising, as their *Mecp2^+/y^* littermates do. This observational datum indicates that *Mecp2^−/y^*-EE are capable of acquiring coordinated movement. We did not detect significant differences in body weight between *Mecp2^−/y^*-EE and *Mecp2^−/y^*-SC mice at the end of the enrichment period (figure 1B).

### Behavioral effects of EE

**Figure 2:**
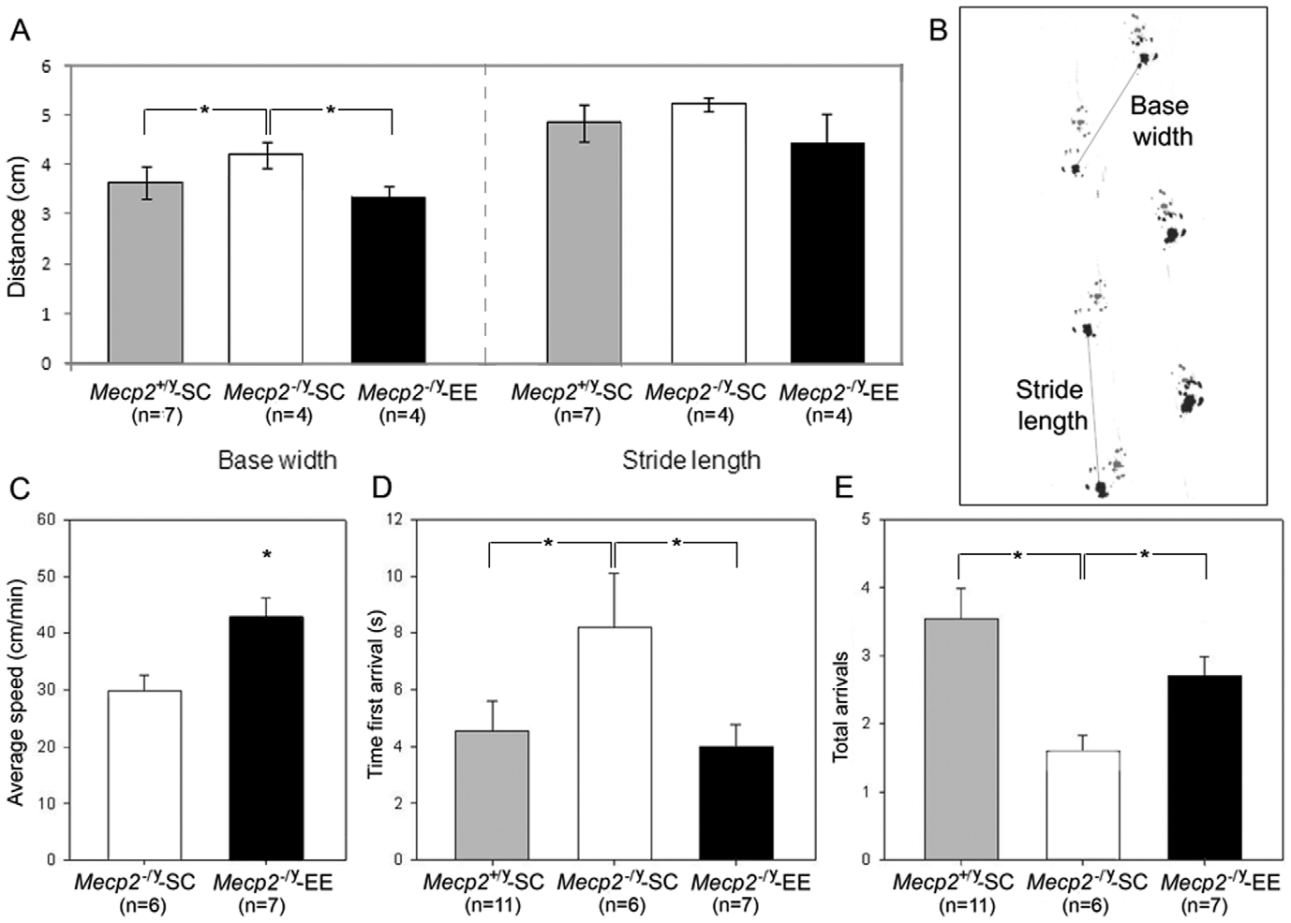
Environmental Enrichment improves neuromotor dysfunction in *Mecp2^−/y^* mice. A, Footprint analysis showed impaired walking patterns in *Mecp2^−/y^*-SC mice compared with *Mecp2^−/y^*-EE and *Mecp2^+/y−^*SC control animals. B, Measures taken for footprint analysis .C, Speed of movement (cm/s) was faster for *Mecp2^−/y^*-EE than *Mecp2^−/y^*-SC mice. D, *Mecp2^−/y^*-EE mice displayed improved motor coordination compared with *Mecp2^−/y^*-SC evidenced by a shorter time to reach the platform for the first time and E, higher number of arrivals to the platform in the elevated beam test. The total number of animals in each group is shown (*n*). Mean ± SEM values are presented. A two-way repeated measures ANOVA (genotype×condition) demonstrated differences in the mean values among the different levels of condition (F = 8.06; *p* = 0.007). Student's t test indicated that the *Mecp2^−/y^*-SC group was significantly different from the other two groups (*: p<0.05).

To substantiate the home cage observation that *Mecp2^−/y^*-EE mice movements were more coordinated than the movements of *Mecp2^−/y^*-SC mice, we compared their gait by a footprint analysis (figure 2B). Comparison of the trail of footprints left by *Mecp2^−/y^*-EE and *Mecp2^−/y^*-SC walking on a straight corridor showed that their stride length and base width was significantly different. Both the hind stride length and hind base width are significantly smaller in *Mecp2^−/y^*-EE mice than in *Mecp2^−/y^*-SC (*N* = 4 each genotype; *p*_stride length_ = 0.053, *p*_hind-base_ _width_ = 0.004; figure 2A). We also analyzed whether EE altered the motor abilities of *Mecp2^−/y^* by means of the open field test and the elevated beam test. Evaluation of the novelty-induced locomotor activity on the open field showed that although the total distance travelled by *Mecp2^−/y^*-EE and *Mecp2^−/y^*-SC was similar (805.7±162.5 and 701.4±124.9 cm for *Mecp2^−/y^*-EE and *Mecp2^−/y^*-SC, respectively), average displacement speed was significantly faster for *Mecp2^−/y^*-EE (42.8±3.5 cm/min) than for *Mecp2^−/y^*-SC (36.5±2.1 cm/min). (p = 0.022, figure 2C). These data suggest that there might be some improvement on the neuromotor dysfunction of *Mecp2^−/y^* mice on EE conditions. Accordingly, we observed that *Mecp2^−/y^*-EE mice outperformed their *Mecp2^−/y^* littermates housed in control conditions in the elevated beam test, in which the mice have to walk on an elevated thin wooden dowel to reach the safety of a partially enclosed platform. *Mecp2^−/y^*-EE mice reached the platform significantly faster than the control *Mecp2^−/y^* mice (figure 2D). In addition, the total number of platform reachings was higher for the *Mecp2^−/y^*-EE (figure 2F). Moreover, the performance of enriched *Mecp2^−/y^* mice was similar to the performance of *Mecp2^+/y^* mice housed either in SC or EE (figure 2D).

**Figure 3:**
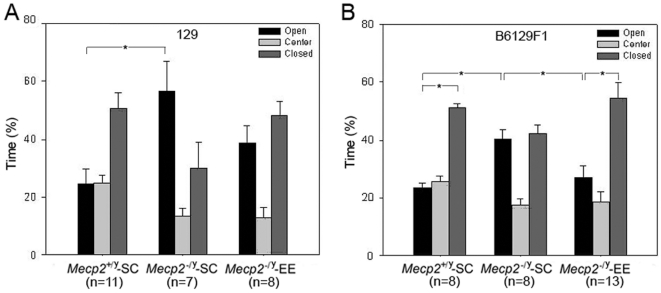
Environmental Enrichment restores a normal behavior in the plus maze test. A, In the elevated plus maze, significant increases in the percentage of time spent in open arms were seen in *Mecp2^−/y^*-SC (129 genetic background) compared with *Mecp2^+/y^*-SC mice. This phenotype is reversed in the *Mecp2^−/y^*-EE mice. B, In a B6129 F1 genetic background, *Mecp2^−/y^*-EE mice showed a behavior indistinguishable from the *Mecp2^+/y^*-SC mice. Mean ± SEM are presented. Two way ANOVA (genotype×condition) demonstrated differences in the mean values among the different levels of Genotype (F = 10.705; p = 0.003) and condition (F = 7.516; p = 0.011). Post hoc Student's t test detected statistical differences between groups, *: p<0.05.

**Figure 4:**
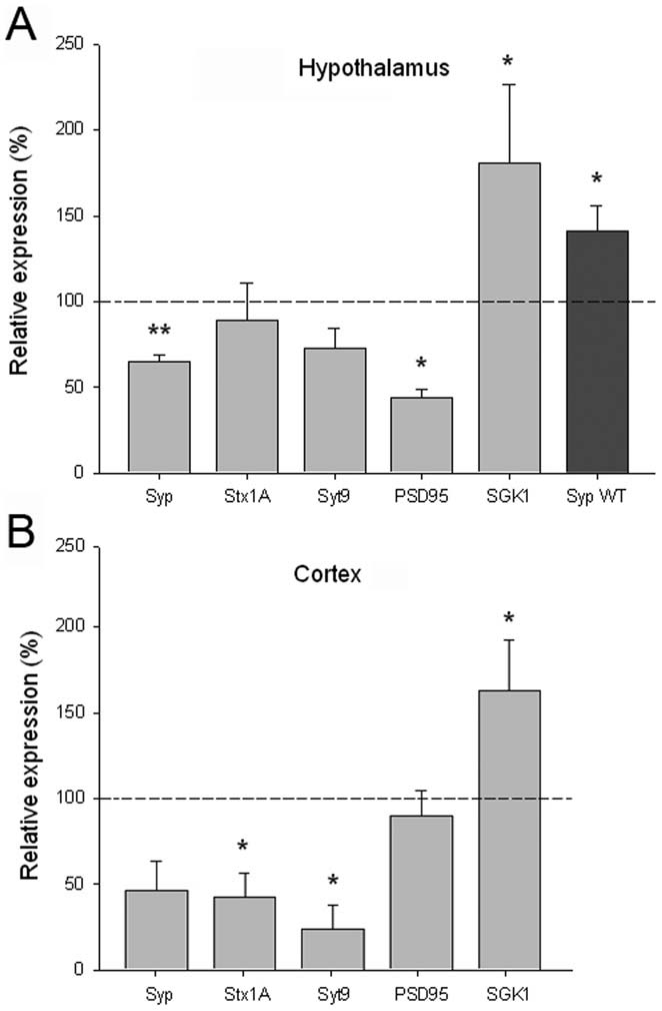
Environmental Enrichment on *Mecp2^−/y^*-EE mice induces a decrease in the expression of a set of synaptic plasticity-related genes. A, A significant difference in the steady-state level of mRNA for Syp, PSD95 and SGK-1 was observed in hypothalamus of *Mecp2^−/y^*-EE in comparison with in *Mecp2^−/y^*-SC mice assessed by qRT-PCR. Syp WT: The expected EE-induced increase in Syp in *Mecp2^+/y^*-EE (normalized to *Mecp2^+/y^*-SC) is shown as a darker bar. B, In samples from cerebral cortex, differences were detected in Syt9, Stx1a and SGK-1. The results are expressed as percentage of the *Mecp2^−/y^*-SC control group. * p<0.05 by Student's test (n = 5 for each group).

*Mecp2^−/y^* mice subjected to tests designed to measure anxiety levels exhibit a behavior consistent with decreased anxiety (see [7] and [26]). Notably, the behavior of the *Mecp2^−/y^*-EE and *Mecp2^−/y^*-SC on the elevated plus maze was significantly different (figure 3). As anticipated by previous reports [7], [26], *Mecp2^−/y^*-SC mice did not show a preference for the safety of the closed arms of the maze as wild type mice do. On the contrary, *Mecp2^−/y^*-SC mice spent significantly more time in the open than in the closed arms (figure 3A). However, if the *Mecp2^−/y^* were housed in an enriched environment, their preference for the open arms disappears (figure 3A). These results indicate that EE prevents the development of this phenotype. To rule out that EE resulted in a stressful condition that increased the levels of anxiety of *Mecp2^−/y^* mice, we measured the expression of *Crh*, a key regulator of the mammalian stress response. We found that the expression of *Crh* in hypothalamus was similar in *Mecp2^−^* ^/y^-EE and *Mecp2^−^* ^/y^-SC mice (figure 4A), suggesting that the restoration of a normal behavior on the plus maze induced by EE in *Mecp2^−^* ^/y^ mice is not just a consequence of elevated stress. Since mice of the 129 genetic background are not very good responders in the plus maze test [27], as evidenced by the behavior of the *Mecp2^+/y^*-SC on this test (figure 3A), we decided to repeat this particular test on a B6129 F1 background. In this genetic background, the preference of *Mecp2^+/y^*-SC for the closed arms of the maze was more pronounced. Again, *Mecp2^−/y^*-SC did not show a preference for the closed arms of the maze, but *Mecp2^−/y^*-EE mice exhibited a behavior indistinguishable from their wild type littermates (figure 3B), further confirming the positive effect of EE on the plus maze phenotype of the *Mecp2^−/y^* mice. Motor performance of the *Mecp2^−/y^* mice was also improved by EE in this background (data not shown)

### Molecular response to EE

Our results strongly suggest that a subset of phenotypic manifestations exhibited by *Mecp2*-null mutants can be ameliorated by environmental stimulation. This implies that EE constitutes a good platform for the identification of neurochemical pathways that could serve as therapeutical targets. As a starting point in this quest, we analyzed the expression of several candidate genes. An increase in the expression of synaptic markers has become a hallmark of the effects of EE. We checked the expression of synaptic markers in 5 week old mice exposed to EE for 14 days. We chose to determine the expression of these markers in hypothalamus and cortex based on the behavioral changes induced by EE in our RTT mouse model and WT mice. We handpicked mice (on a 129 genetic background) that displayed a visually attenuated phenotype for this analysis. Surprisingly, we found that expression of postsynaptic density-95 (PSD95) and synaptophysin (Syp) was significantly downregulated in hypothalamus of *Mecp2^−^* ^/y^ mice under EE conditions (figure 4A). The steady state level of PSD95 and Syp mRNAs in *Mecp2^−/y^*-EE, relative to *Mecp2^−/y^*-SC, in the hypothalamus was 43.2±2.7 and 64.7±4.0%, respectively (p<0.05). A similar trend was observed for synaptotagmin (Syt9, 72.9±11.3%) and activity regulated cytoskeletal-associated protein (Arc, 48.5±17.2) although it did not reach statistical significance (p = 0.07 and 0.057, respectively). These data contrast with the observed upregulation of synaptophysin in the littermate wild type controls housed together with the *Mecp2^−/y^*-EE and therefore subjected to the exact enriched environment (*Mecp2^+/y^*-EE, figure 4B). Interestingly, analysis of candidate gene expression in the cortex showed that *Mecp2^−/y^*-EE mice have reduced expression of syntaxin 1A (Stx1a, 42.8±13.7%,), synaptotagmin (23.6±13.7%) and a trend for diminished expression of synaptophysin (46.5±16.8% p = 0.06), as compared to expression levels of *Mecp2^+/y^*-SC (figure 4B). The EE-induced reduction in syntaxin1A is notable, since it has been reported that non enriched *Mecp2^−/y^* already exhibit reduced expression of this gene [16]. However, we did not replicate this finding; the expression of Stx1A was similar in our *Mecp2^+/y^* and *Mecp2^−/y^* in standard conditions (data not shown). We could not detect significant changes in candidate gene expression induced by EE in midbrain of *Mecp2^−/y^* mice.

## Discussion

Elucidating the factors that enable RTT patients to favorably change the course of the disease is a relevant task. Animal models are increasingly important in addressing this issue. Importantly, the availability of mouse models of RTT has allowed researchers to determine that the dysfunctionality caused by lack of MeCP2 is not irrevocable. Guy et al. [28] and Giacometti et al. [29] proved that the RTT-like symptoms of *Mecp2* null mice could be fully reversed by conditionally activating a functional copy of MeCP2.These data provide hope for phenotypic amelioration in RTT, but do not provide immediate therapeutic strategies since attaining appropriate MeCP2 expression throughout the CNS is not feasible right now because of the challenges in delivering the correct MeCP2 dosage only to neurons that lack it, given that the slightest perturbation in MeCP2 level seems deleterious. In fact, we [30] and Giacometti et al. [29] demonstrated that postnatal reactivation of MeCP2 in mutant animals is not sufficient to rescue the complete null phenotype unless MeCP2 levels are restored to the expected wild-type levels and done so in a sufficient number of neurons. A recent paper [31], indicating that glial cells lacking MeCP2 have negative effects on the growth of neuronal dendrites implies that for a full phenotypic rescue, restitution of MeCP2 should also include non neuronal cells.

In addition, it has been shown that exposure to an enriched environment was able to modify the disease course in mouse models of RTT. Kondo et al. [24] reported that *Mecp2* ^+/*−*^ females, but not males, housed in enriched conditions were spared the motor discoordination exhibited by control *Mecp2* ^+/*−*^ females. This group subjected the mice to EE from 4 weeks of age until the end of the study (at 10 weeks for males and 30 weeks for females). Thus, the authors proposed that a functional copy of *Mecp2* was necessary for benefiting from EE. On the other hand, Nag et al. [25] showed that EE, starting at weaning age, precluded the development of hypoactivity in *Mecp2* null males.

In this study we show that *Mecp2^−^* ^/y^ mice housed from 3 to 5 weeks of age in an enriched environment that favors social interaction, exercise and exploratory activities display an attenuated neuromotor phenotype. *Mecp2^−/y^*-EE mice are distinguishable from *Mecp2^−/y^*-SC mice by a trained observer, they are more active, their movements are more coordinated and have in general a healthier look. *Mecp2^−/y^*-EE mice are as capable as wild type mice of traversing a thin elevated beam, a task that requires good motor coordination. In addition, *Mecp2^−/y^* mice moved faster and the ataxic gait was observably improved in conditions of EE.

Further, we observed that the behavior of the *Mecp2^−/y^*-EE mice on the plus maze was similar to the behavior exhibited by wild type mice, in contrast to *Mecp2^−/y^*-SC mice that behaved as if they have decreased anxiety. However, the *Mecp2^−/y^*-SC data (i.e., increased time spent in the open arms of the plus maze) could be alternatively interpreted as resulting from abnormal perception of the environment, as if the mutant mice are not completely able to perceive the danger of the unprotected walkway instead of a reflection of decreased anxiety. We favor this interpretation, based on the reports of elevated anxiety observed in RTT patients and the abnormal expression of genes that are induced during the stress response by glucocorticoids [32], [33]. Thus we propose that EE restores MeCP2 mutant's ability to appropriately perceive environmental clues.

Our data support the notion that EE improves phenotypic manifestations of mouse models of RTT [24], [25]. Notwithstanding differences in the mouse models utilized (*Mecp2^tm1Tam^* [24], *Mecp2^1lox^* [25] or *Mecp2^tm1.1Bird^* [this report]), the enrichment paradigms (continuous from 4 weeks of age [24], continuous from 3 weeks of age [25] or transient from 3 to 5 weeks of age [this report]) and the methods used to test its effects, EE is capable of eliciting improvement in some RTT like phenotypes.

The EE-induced phenotypic amelioration suggests that it could constitute a good platform to identify critical neurobiologic alterations that underlie phenotypic improvements in RTT mouse models. It has been postulated that the beneficial effects of EE are mediated largely by modulation of expression of a number of genes involved in neuronal structure, synaptic signaling and plasticity [34]–[36]. We thus compared the expression of synaptic markers in *Mecp2*-null mice subjected to EE or SC. Surprisingly, we observed decreased expression of synaptic proteins in brains of *Mecp2^−/y^*-EE mice. Synaptophysin and PSD95 were downregulated in the hypothalamus and syntaxin 1a and synaptotagmin were significantly decreased in the cortex of *Mecp2^−^* ^/y^ mice under EE conditions. On the contrary, control wild type mice subjected to EE showed the expected elevation in synaptophysin levels. In addition to a diametrically opposed response of *Mecp2^−^* ^/y^ mice to what was expected based on previous studies of EE transcriptional effects with respect to synaptophysin, syntaxin 1a and synaptotagmin, we also did not detect changes in gene expression of genes shown to respond to EE such as IGF-I, IGFbp, BDNF, Grin2B, Gria, Gap43, Nefm, Dyn1 and N-Cadh in brains of *Mecp2^−/y^*-EE mice. It is also notable that in spite of an observable EE-induced motor improvement in *Mecp2^−^* ^/y^ mice, we did not detect EE-induced changes in gene expression in midbrain, suggesting that the motor amelioration could be mediated by brain regions different from midbrain structures.

The dynamics of synaptic connectivity is determined by a balance between synapse formation and elimination that initiates in development and continues into adulthood [37]. It has been shown that the control of synapse number is subverted in mice lacking MeCP2. At 2 weeks of age, *Mecp2^−^* ^/y^ mice have a reduced number of glutamatergic synapses that is compensated by 5 weeks of age when housed in standard conditions [13]. The EE-induced decrease in mRNA levels for synaptic proteins in *Mecp2^−^* ^/y^ mice suggests a reduction in synapse number. Since our EE program started when mice were of 3 weeks of age, we cannot differentiate whether the results reflect an interference in the process that culminates with synapse number compensation or a loss of synapses during EE. Nevertheless, our data strengthen the hypothesis of alterations in synaptic function underlying the pathophysiology of Rett syndrome.

Although the relevance of these molecular findings to the partial behavioral amelioration induced by EE is yet unknown, they indicate that neurons lacking MeCP2 are unable to respond to EE through canonical pathways.

**Note Added in Proof**: While this manuscript was under review, Lonetti et al. (Biol Psychiatry. 2010, 67:657–65.) reported that early and continuous EE (starting at postnatal day 10) improved motor coordination and motor learning in *Mecp2* ^−/y^ male mice. They also show that this EE paradigm induced alterations in synaptic density, LTP, and augmented BDNF levels.
